# Lung protection by inhalation of exogenous solubilized extracellular matrix

**DOI:** 10.1371/journal.pone.0171165

**Published:** 2017-02-02

**Authors:** Jinglei Wu, Priya Ravikumar, Kytai T. Nguyen, Connie C. W. Hsia, Yi Hong

**Affiliations:** 1 Department of Bioengineering, University of Texas at Arlington, Arlington, Texas, United States of America; 2 Joint Graduate Program in Biomedical Engineering between University of Texas at Arlington and University of Texas Southwestern Medical Center, Dallas, Texas, United States of America; 3 Department of Internal Medicine, Pulmonary and Critical Care Medicine, University of Texas Southwestern Medical Center, Dallas, Texas, United States of America; Ohio State University, UNITED STATES

## Abstract

Decellularized extracellular matrix (ECM) contains complex tissue-specific components that work in concert to promote tissue repair and constructive remodeling and has been used experimentally and clinically to accelerate epithelial wound repair, leading us to hypothesize that lung-derived ECM could mitigate acute lung injury. To explore the therapeutic potential of ECM for noninvasive delivery to the lung, we decellularized and solubilized porcine lung ECM, then characterized the composition, concentration, particle size and stability of the preparation. The ECM preparation at 3.2 mg/mL with average particle size <3 μm was tested *in vitro* on human A549 lung epithelial cells exposed to 95% O_2_ for 24 hours, and *in vivo* by tracheal instillation or nebulization into the lungs of rats exposed intermittently or continuously to 90% O_2_ for a cumulative 72 hours. Our results showed that the preparation was enriched in collagen, reduced in glycosaminoglycans, and contained various bioactive molecules. Particle size was concentration-dependent. Compared to the respective controls treated with cell culture medium *in vitro* or saline *in vivo*, ECM inhalation normalized cell survival and alveolar morphology, and reduced hyperoxia-induced apoptosis and oxidative damage. This *proof-of-concept* study established the methodology, feasibility and therapeutic potential of exogenous solubilized ECM for pulmonary cytoprotection, possibly as an adjunct or potentiator of conventional therapy.

## Introduction

Acute lung injury (ALI) has an incidence from 16 per 100,000 among youths to 306 per 100,000 in the elderly, with 200,000 cases occurring in the United States annually and an in-hospital mortality of 40% [1,2]. Regardless of the specific cause of ALI, the common manifestations include increased permeability of the epithelium and endothelium, as well as recruitment and activation of alveolar macrophages and neutrophils to the lung, which lead to the release of pro-inflammatory and cytotoxic mediators [3]. Furthermore, the increased reactive oxygen species (ROS) damage cell components including proteins, lipids, carbohydrates and DNA, promote apoptosis and reduce endogenous antioxidant capability [4–7].

Various interventions have been attempted to reduce ROS production and/or increase antioxidant production in the lung. Surfactant protein-D evoked antioxidant expression and alleviated proinflammatory cytokine production in hyperoxia-exposed lung [8]. The administration of vascular endothelial growth factor prevented alveolar damage from hyperoxia [9]. Epidermal growth factor-like domain 7 was secreted by vascular endothelial cells and highly expressed in lung, and it was found to protect endothelial cells from hyperoxia-induced cell death by inhibiting mitochondria-dependent apoptosis pathway [10]. Although the above and other agents, including nitric oxide, glucocorticoids and lysofylline, have been used to alleviate ALI, none has been able to decrease mortality [11]. There remains an urgent need to explore novel strategies to reduce tissue damage and improve outcome in ALI.

Recently, decellularized extracellular matrix (ECM) has been extensively studied for tissue regeneration, and organ replacement [12]. Decellularized whole lung ECM scaffold composed of collagen, elastin, fibronectin, laminin and glycosaminoglycans (GAGs) facilitated lung cell growth *in vitro* and has been implanted into the rat thorax [13,14]. Tissue-specific ECM has been shown to accelerate cell proliferation in vitro and promote organ remodeling [15–18]. Although the mechanism of ECM-mediated tissue remodeling remain incompletely understood, it is believed that coordinated interactions among the complex ECM components modulate key cellular functions such as proliferation, migration and differentiation [19,20] while the peptide fragments released from ECM favorably modulate host immune response [21,22]. In one study, tracheal instillation of decellularized powder of urinary bladder ECM promoted pulmonary epithelial cell chemotaxis, migration, and repair in bleomycin-induced pulmonary fibrosis [23].

We hypothesized that decellularized lung-derived ECM protects lung cells and intact lungs from oxidative damage, and is a potential treatment option for ALI. As *proof-of-concept*, we solubilized the decellularized porcine lung ECM in a novel formulation of solution and fine microparticle suspension suitable for nebulization and non-invasive inhalational delivery to the lung. We characterized this formulation, and then assessed its cytoprotective effects *in vitro* in lung epithelial cells and *in vivo* in a rodent model of ALI.

## Materials and methods

### Materials

Sodium dodecyl sulfate (SDS), hydrochloric acid (HCl), sodium hydroxide (NaOH), phosphate-buffered saline (PBS), phenol/chloroform/isoamyl alcohol, chloramine T/oxidation buffer, dimethylaminobenzaldehyde, pepsin, urea, thiourea, dithiothreitol, 3-(4,5-dimethylthiazol-2-yl)-2,5-diphenyltetrazolium bromide (MTT) and fetal bovine serum (FBS) were obtained from Sigma-Aldrich, St. Louis, MO. Dulbecco’s Modified Eagle Medium (DMEM)/F12, penicillin and streptomycin were purchased from Life Technologies, Inc. (Carlsbad, CA).

### Decellularization of porcine lung

**Fig 1:**
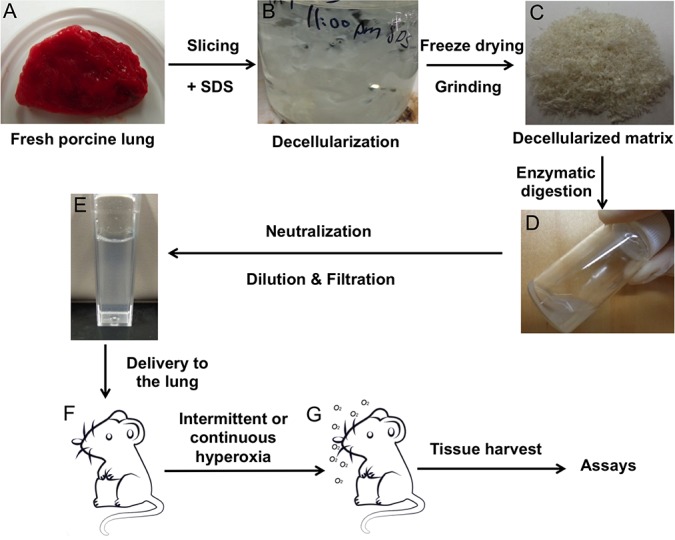
Lung ECM solution preparation and delivery. Porcine lung (A) was sliced and treated with SDS (B) to obtain decellularized ECM (C). The lung ECM was enzymatically digested with pepsin (D), followed by neutralization and dilution, as well as filtration through a 1.2 μm filter to yield the ECM solution/suspension (E). Rats received the lung ECM preparation (F) followed by hyperoxia exposure and tissue analysis (G).

The decellularization procedures are illustrated in Fig 1. Porcine lungs were freshly harvested from adult pigs (body weight 80–100 kg) from a local slaughterhouse (Fig 1A). Decellularization was performed by modifying a previously reported method [24]. Briefly, the lungs were cut into thin slices (1 mm) and washed with deionized (DI) water five times to remove excess blood. The slices were treated with 0.5% SDS solution under constant stirring for 72 h (Fig 1B). The SDS solution was refreshed every 24 h. Then the slices were rinsed with a large amount of DI water to remove residual SDS, and frozen at -80°C, following by freeze-drying to obtain decellularized lung ECM. The decellularized ECM was ground into coarse powders (Fig 1C) and stored at -20°C for future use.

### Characterization of decellularized ECM

Histology of the decellularized lung ECM was compared to that of the native porcine lung fixed with 4% paraformaldehyde at a hydrostatic pressure of 25 cm H_2_O. The samples were sectioned (4 μm) and stained with hematoxylin and eosin (H&E). The slices of decellularized lung ECM before grinding and native lung tissue were freeze-dried, sputter coated with silver and observed under a scanning electron microscope (SEM, S-3000N, Hitachi).

Composition of ECM was analyzed by mass spectrometry [25]. Briefly, ECM sample was solubilized in a solution of 5 M urea, 2 M thiourea, 50 mM dithiothreitol and 0.1% SDS under constant stirring for 2 days. The sample was then analyzed by a high performance liquid chromatography ion trap time of flight mass spectrometry (LCMS IT-TOF, Shimadzu Scientific Instruments, Kyoto, Japan) equipped with electrospray ionization (ESI) ion source at the Shimadzu Center of the University of Texas at Arlington. A Shimadzu Shim-pack MAYI-ODS (4.6 mm i.d. × 10 mm L, 3 μm d_p_) column and a Halo C18 column (2.1 mm i.d. × 100 mm L, Phenomenex, Torrance, CA) were used as a trap column and an analytical column, respectively. The mobile phase compositions were H_2_O/acetonitrile (95/5) and acetonitrile/H_2_O (95/5) for mobile phase A and mobile phase B, respectively. Both phases contained 0.1% formic acid and kept a constant flow rate at 0.25 mL/min. The sample was injected into the trap column at 0.6 mL/min using a pump, and then at 0.05 mL/min after the system configuration was switched from phase A to phase B. The LCMS IT-TOF system was used in the automatic mode with a scan range of 150–600 (m/z) and an ion accumulation time of 56 msec. The heating block and curved desolvation line were kept at 250°C and 300°C, respectively. The ESI source voltage was set at 4.5 kV. A LCMSsolution (version 3.5) software was utilized for result analysis.

### DNA content

Following established procedures [26], the ECM was digested using pepsin (1 mg/mL) in 0.01 M HCl solution and centrifuged at 2980 g for 10 min to remove protein remains. DNA was extracted by centrifuging the supernatant at 10,000 g in phenol/chloroform/isoamyl alcohol (25:24:1) for 30 min. Then DNA in the aqueous layers of the centrifuged samples was precipitated using 3 M sodium acetate and ethanol mixed solution (v/v = 1:20) at –20°C overnight. The extracted DNA was dehydrated in a vacuum oven overnight and rehydrated in 1X Tris-EDTA buffer (Invitrogen, Life Technologies, Inc. Carlsbad, CA). Finally, DNA contents were determined using a PicoGreen DNA assay (Life Technologies, Inc. Carlsbad, CA) (n = 3). The DNA content of native tissue was measured using the same method for comparison.

### Collagen content

Total collagen content was assessed by measuring hydroxyproline, which makes up 14.3% of collagen by weight [27]. Briefly, the decellularized ECM and the native lung tissue were hydrolyzed with 6 M HCl at 120°C for 3 h and then dehydrated at 65°C. Each sample was mixed with 100 μL of chloramine T/oxidation buffer mixed solution and incubated at room temperature for 5 min. Then 100 μL dimethylaminobenzaldehyde solution was added to each sample and incubated at 60°C for 90 min. The sample absorbance was read at 560 nm on an Infinite M200 plate reader (Tecan, UK) (n = 3), and a standard curve was generated from a series of samples of known hydroxyproline concentrations.

### GAG content

Decellularized ECM and native lung tissue were digested in 1 mg/mL pepsin buffered in 0.01 M HCl solution. The GAG contents were determined using Blyscan Sulfated Glycosaminoglycan Assay (Biocolor, UK) according to the manufacture instruction (n = 3). Briefly, the digests were incubated with Blyscan dye reagent for 30 min and then centrifuged at 12,000 rpm for 10 min. Platelets were collected and decomposed with Blyscan dissociation reagent, followed by centrifuging at 12,000 rpm for 5 min to remove foam. Then the samples were read at 565 nm using an Infinite M200 plate reader. Pepsin in 0.01 M HCl solution was used as a negative control and its reading was subtracted from the all measured values.

### Preparation and characterization of decellularized ECM suspension/solution

Decellularized ECM (15 mg/mL) was digested in pepsin solution (1 mg/mL in 0.01 M HCl) under constant stirring at room temperature until no visible particle was observed (Fig 1D). The digest was neutralized by adding 1/10 digest volume of 0.1 M NaOH and 1/9 final volume of 10X PBS, and then diluted to ECM concentrations of 2, 3, 4 and 5 mg/mL using 1X PBS in an ice bath (Fig 1E). The diluted solution was passed through a 1.2 μm filter to obtain a fine ECM suspension/solution. Particle size in the ECM solution was characterized using dynamic light scattering (DLS, Brookhaven Instruments Corporation, NY). One microliter of ECM solution was dried in an oven at 110°C for 6 h and the residual dry powder was weighed (n = 4). One microliter of 1X PBS solution was dried and weighed, and then subtracted from the weight of the above mentioned dry powder to determine the ECM content in the solution (n = 4). To visualize ECM particle morphology, one droplet of the ECM solution was coated on a piece of conductive tape and freeze-dried overnight. Then the sample was sputtered coated with silver and observed under a scanning electron microscope (SEM, S-3000N, Hitachi, Japan).

### Cell culture and hyperoxia challenge

Human lung epithelial cells (A549) (American Type Culture Collection, Manassas, VA) were cultured in DMEM/F12 with 1% L-glutamine supplemented with 10% FBS, 100 U/mL penicillin and 100 μg/mL streptomycin in a humidified incubator at 37°C in 5% CO_2_. A total of 10,000 cells were seeded in each well of a 24-well plate and cultured with DMEM/F12 complete medium. When the cells reached 80% confluence, the medium was replaced with DMEM/F12 complete medium supplemented with ECM preparation at concentrations of 0.15, 0.3 and 0.45 mg/mL. Control cells were cultured on tissue culture polystyrene (TCPS) with DMEM/F12 complete medium without ECM supplement. The samples were exposed to either normoxia (21% O_2_/5% CO_2_) or hyperoxia (95% O_2_/5% CO_2_) for 24 h at 37°C. The hyperoxia environment was built using a modulator incubator chamber (Billups-Rothenberg, CA). Cell viability was assessed by MTT assays (n = 3). The absorbance was recorded at 490nm on an Infinite M200 plate reader (Tecan, UK). A live (calcein-AM)/dead (ethidium homodimer-1) assay (Life Technologies, Inc. Carlsbad, CA) was used to stain live and dead cells. The stained cells were imaged with a microscope (Eclipse *Ti*, Nikon, Japan), and the numbers of live cells and dead cells were counted using Image J (National Institute of Health, USA). The cell survival ratio (%) was calculated as the number of live cells/the number of total cells ×100% (n = 3).

### Animal studies

All animal protocols were conducted following the National Institutes of Health Guide for the Care and Use of Laboratory Animals, and approved by the Institutional Animal Care and Use Committee at the University of Texas Southwestern Medical Center.

### Inhalational delivery

Sprague-Dawley rats (body weight ~300 g) were anesthetized (intraperitoneal injection of ketamine 50 mg/kg and xylazine 5 mg/kg) and intubated (14-gauge cannula). Heart rate and transcutaneous O_2_ saturation were monitored via a tail cuff (Kent Scientific, Torrington, CT). The ECM solution (3.2 mg/mL, 1.5 mg) or saline (n = 4 each) was delivered to the lungs via one of two protocols: a) Direct instillation of ECM or saline into the trachea. The animal was rotated from side to side several times to ensure even distribution of the solution throughout the lung. Immediately following recovery from anesthesia, the animal was exposed to continuous hyperoxia (90% inspired O_2_) in an environmental chamber (Biospherix™, Lacona, NY) for 3 days. b) Nebulization of ECM or saline through the tracheal cannula. ECM solution suspended in 0.5 ml of sterile saline was sonicated for 2 min (300VT ultrasonic homogenizer, Biologics, Manassas, VA), immediately aerosolized using a vibrating mesh nebulizer (4–6 μm droplets, Aeroneb™, Aerogen, Galway, Ireland), and delivered via the tracheal cannula over ~3 min. Following recovery from anesthesia the animal was exposed to intermittent hyperoxia (90% O_2_ for 24 h) alternating with normoxia (21% O_2_ for 24 h) for a total of 3 hyperoxia-normoxia cycles over 6 days. During hyperoxia, expired carbon dioxide was scrubbed by soda lime. Body weight was monitored daily. Additional age-matched animals were maintained in normoxia as simultaneous untreated controls.

### Lung harvest

At the end of exposure, animals were anesthetized and intubated as described above, then killed by an intraperitoneal overdose injection of pentobarbital (86 mg/kg) and phenytoin (11 mg/kg). The left lung was removed and flushed clear of blood. Samples were taken and snap frozen in liquid nitrogen. The right lung was fixed by tracheal instillation of 4% paraformaldehyde at 25 cm H_2_O of airway pressure.

### Injury assessment

Apoptosis: Caspase-8 activity was measured by colorimetric assay (ApoAlert™, Clontech, Mountain View, CA). DNA damage: DNA was extracted using DNAzol™ (Life Technologies, Grand Island, NY), precipitated in 100% ethanol, washed with 70% ethanol and suspended in 8 mM NaOH, The 8-hydroxy-2’-deoxyguanosine (8-OHdG) level was measured by ELISA (OxiSelect™, Cell BioLabs). Protein oxidation: Protein Carbonyl level was measured by ELISA (OxiSelect™ Cell BioLabs). Lipid oxidation: 8-isoprostane level was measured by EIA (Cayman Chemical, Ann Arbor, MI). Edema estimation: Lung tissue (~100 mg) was weighed and transferred to a platinum ashing crucible on a hot plate (100°C) under a heat lamp for 2h. The sample-containing crucible was weighed to determine the sample dry weight. The crucible was placed overnight in the oven (600°C) and the ash weight determined. The ash was dissolved in 2 mL of HCl and the sodium content measured by flame photometry. Sodium-to-dry weight ratio is a surrogate marker for the relative amount of interstitial fluid (extracellular/intracellular sodium concentration: 135-140/10-15 mM). Assays were performed in triplicates.

### Statistical analysis

All data were expressed as mean ± standard deviation. Statistical analysis was performed using Statview™ software. The values of DNA, collagen and GAG contents were analyzed using Student’s *t*-test. The statistical analyses of the *in vitro* results were performed with one-way analysis of variance (ANOVA), followed by a Tukey post-hoc test. The *in vivo* results were compared across conditions by two-way ANOVA with post-hoc test by Fisher’s protected least significant difference method. P<0.05 was considered significant.

## Results

### Characterization of the decellularized lung ECM

**Fig 2:**
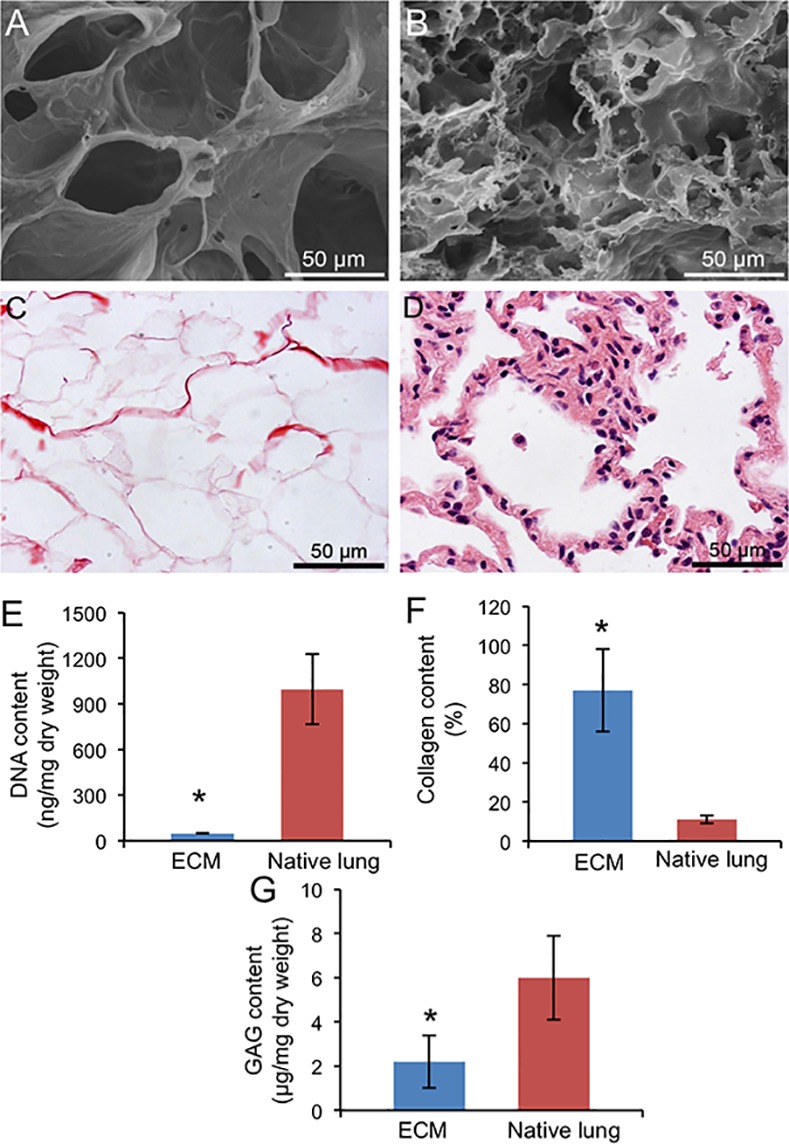
**Characterization of lung ECM by SEM (A and B) and H&E staining (C and D).** Images of decellularized lung ECM (A and C) were compared to that of the native lung (B and D). Quantitative analysis of DNA (E), collagen (F) and GAG (G) contents in decellularized lung ECM and native lung tissue. Mean ± SD. P < 0.05: * vs. native lung tissue.

Scanning electron microcopy (SEM) images and histologic sections demonstrate the overall integrity of alveolar architecture and an absence of cell nuclei in decellularized lung ECM (Fig 2A and 2C) compared to native lung tissue (Fig 2B and 2D). DNA content in lung ECM (49 ± 4 ng/mg dry weight, Fig 2E) was 5% of that in the native lung (996 ± 231 ng/mg), indicating adequate decellularization. Decellularized lung ECM had a significantly higher collagen content (77 ± 21%) than that of the native lung (12 ± 2%) as shown in Fig 2F. Sulfated GAG content (2.2 ± 1.2 μg/mg dry weight) was significantly lower than that in the native lung (6.0 ± 2.0 μg/mg) (Fig 2G). Mass spectrometry revealed the complex bioactive components of lung ECM including nuclear factor kappa-B (NF-κB), NADH dehydrogenase and epidermal growth factor-like domains protein (Table 1).

**Table 1:** Mass spectrometry analysis of decellularized lung ECM.

Full name	Accession No.	Protein score
Collagen alpha-5(IV)	XP_004022477	144
Collagen alpha-5(IV)	XP_004022478	144
NADH dehydrogenase	BAK19372	190
Nuclear factor NF-kappa-B	XP_004009716	174
Chromodomain-helicase-DNA-binding protein	XP_003757294	155
ATP-binding cassette sub-family G member 4-like	XP_001917470	144
Multiple epidermal growth factor-like domains protein	XP_002924008	143
NFKB1 protein	AAI53233	106
Copper-transporting ATPase	XP_596258	115
Cytochrome c oxidase subunit I	YP_004935412	165
Neutralized-like protein 4	XP_002718856	136
Sodium/glucose cotransporter 4	ELK06136	97
Acetylcholine receptor	ELK17105	135
Retrotransposon gag domain-containing protein 1	XP_004000841	146
Type III secretion translocator protein	YP_004651318	156
Glutamate synthase	WP_011645127	144
Fatty acid synthase	YP_00686574	170
Carbohydrate binding family protein	YP_004319456	143
PE-PGRS family protein	YP_001850993	141
GA27210, isoform A	XP_002137435	137

### Decellularized lung ECM solution

**Fig 3:**
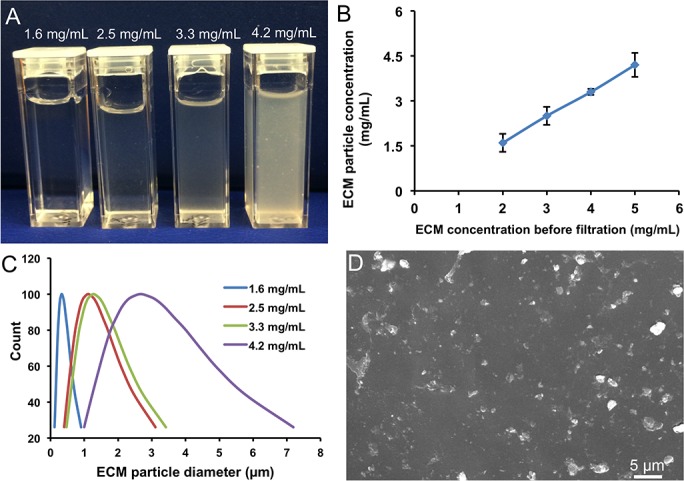
Characterizations of lung ECM solution/suspension. (A) Photos show decreasing transparency of the solution/suspension with increasing ECM concentrations from 1.6 to 4.2 mg/mL after filtration. (B) ECM particle concentration in solution/suspension before and after filtration. (C) Particle size distribution of the filtered lung ECM preparation. (D) Particle morphology after filtration from 4 mg/mL is shown by SEM.

Lung ECM was processed into solution through enzymatic digestion and filtration. The final filtered solutions (Fig 3A) appeared transparent to translucent to cloudy with increasing ECM concentration (1.6 ± 0.3 to 4.2 ± 0.4 mg/mL) (Fig 3B). The yields after filtration were approximate 80%. The 1.6 mg/mL ECM solution showed the narrowest particle diameter distribution (from 120 to 900 nm, Fig 3C). Particle diameter distribution at higher ECM concentrations ranged from 0.4 to 3 μm (2.5 and 3.3 mg/mL solution) and 1 to 7 μm (4.2 mg/mL solution). To ensure smooth instillation and the maximal ECM uptake *in vivo*, we selected the ECM solution with a post-filtration particle concentration of 3.2 ± 0.2 mg/mL and an average particle size less than 3 microns (Fig 3C and 3D) for further *in vivo* study.

### Protection against hyperoxia challenge in human lung epithelial cells

**Fig 4:**
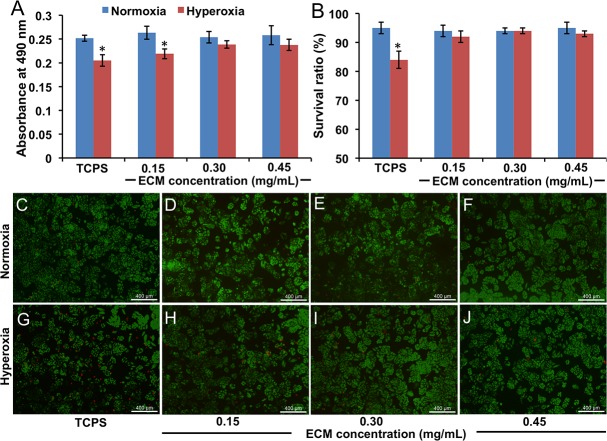
*In vitro* cytoprotective evaluation of lung ECM solution/suspension. (A) A549 cell viability denoted by absorbance intensity at 490 nm using MTT assay. (B) A549 cell survival ratio was determined by live/dead staining. P < 0.05: * vs. TCPS control. (C to J) Live/dead staining images of A549 cells on TCPS in mediums without lung ECM (C and G) and with 0.15 (D and H), 0.3 (E and I) and 0.45 (F and J) mg/mL lung ECM under normoxia (C, D, E and F) and hyperoxia (G, H, I and J).

After 24 h of hyperoxia challenge, human lung epithelial (A549) cells cultured on TCPS without lung ECM exhibited significantly reduced cell viability compared with the corresponding normoxia control (Fig 4A). Similarly decreased viability under hyperoxia was also observed in the cells treated with a low ECM concentration (0.15 mg/mL). In comparison, higher ECM concentrations (0.3 and 0.45 mg/mL) preserved cell proliferation in hyperoxia compared to normoxia. Cell survival ratios assessed by live/dead staining (Fig 4B–4J) demonstrated a similar trend. In the absence of lung ECM, A549 cells showed a significantly decreased survival ratio in hyperoxia compared with normoxia. No significant difference between the hyperoxia and normoxia conditions was observed in the lung ECM-treated groups (Fig 4B) (p > 0.05).

### Protection against hyperoxic lung damage

**Fig 5:**
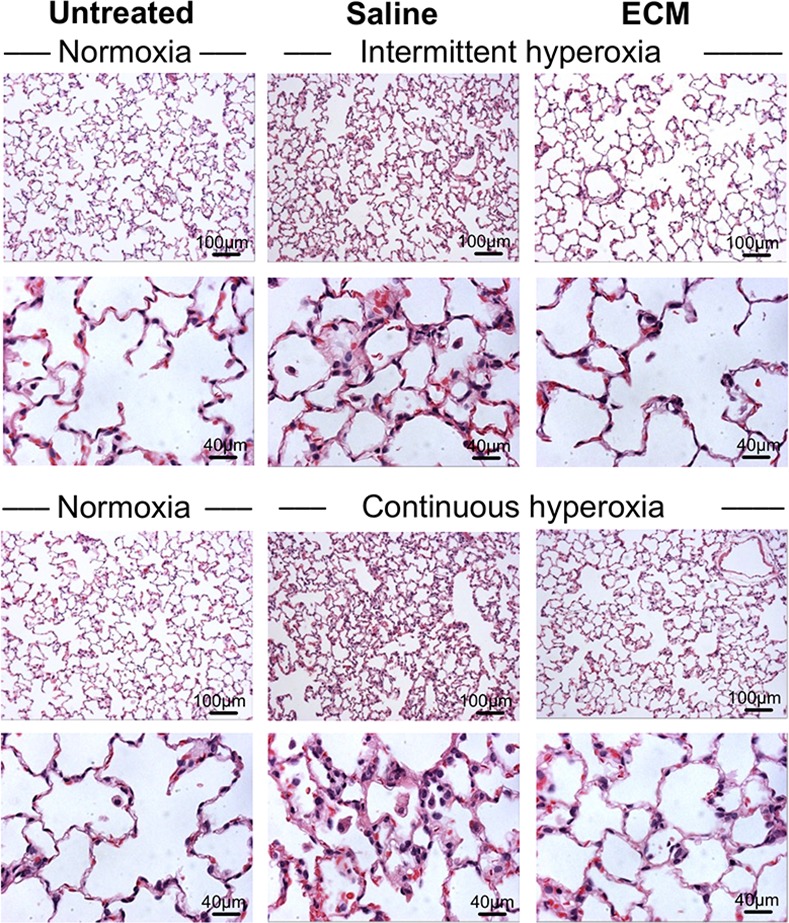
Lung morphology (H&E) of rats receiving either tracheal instillation of ECM solution/suspension followed by continuous hyperoxia (90% O_2_ × 3 d, top two rows) or nebulization of ECM solution/suspension followed by intermittent hyperoxia (90% alternating with 21% O_2_ every 24 h over a total of 6 d, lower two rows) compared to that in corresponding saline-treated hyperoxia-exposed animals as well as in untreated control animals exposed to normoxia (21% O_2_).

**Fig 6:**
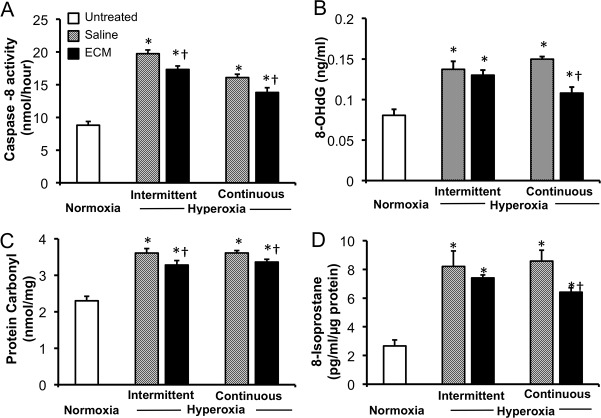
Effects of lung ECM treatment on apoptosis and oxidative tissue damage in rats exposed to intermittent or continuous hyperoxia compared to saline treatment in hyperoxia-exposed and untreated normoxic control animals: caspase-8 activity (A), 8-OHdG (B), protein carbonyl (C) and 8-isoprostane (D). Mean ± SD. p < 0.05: * vs. normoxia control; † vs. saline treatment in the same exposure group.

In rats receiving tracheal instillation of lung ECM solution followed by either continuous or intermittent hyperoxia challenge compared to those receiving saline only, the expected hyperoxia-induced alveolar septal thickening, tissue edema and cell/fluid exudation were significantly reduced as assessed by morphology (Fig 5). Compared to the corresponding saline-treated controls, ECM treatment significantly attenuated apoptosis (caspase-8 activity) during continuous and intermittent hyperoxia by 14–18% (Fig 6A), oxidative DNA damage (8-OHdG) during continuous but not intermittent hyperoxia (28% and 5%, respectively) (Fig 6B), oxidative protein damage (carbonyl) during continuous and intermittent hyperoxia (~8%) (Fig 6C), and lipid oxidative damage (8-isoprostane) during continuous but not intermittent hyperoxia (25% and 9%, respectively) (Fig 6D).

## Discussion

Most studies of decellularized ECM focused on preserving the whole lung ECM scaffold for re-cellularization and organ replacement. To our knowledge, this is the first study to develop a decellularized lung-derived ECM suspension/solution suitable for inhalational delivery to the lung, and test its effects on injury mitigation. Our major findings are as follows: By enzymatic digestion and filtration, porcine lung ECM was processed into a fine solution/suspension with particle diameters less than 5 μm, making it suitable for nebulization to reach the distal lung. The ECM preparation significantly increased viability of lung epithelial cells exposed to hyperoxia *in vitro*. A dilute ECM preparation (3.2 mg/mL concentration) was delivered into rat lung by either tracheal instillation or nebulization. Both delivery methods showed similar ameliorative effects against acute lung damage induced by either continuous or intermittent hyperoxia exposure. These data established the feasibility a novel ECM-based formulation for nebulization and non-invasive targeted delivery to the lung, and demonstrated its beneficial effects in alleviating acute lung injury.

Decellularized ECM has been utilized for investigation of lung tissue repair and regeneration [13,28]. Native lung ECM harbors a complex mixture of endogenous growth factors, proteoglycans and bioactive molecules [29,30] including those detected by us (**Table 1**) and reported by others [28,31,32]. These bioactive ECM components interactively modulate integrated responses to physiological and pathophysiological challenge. Decellularized ECM has also been investigated for disease treatment. For example, tracheal instillation of decellularized ECM powder from urinary bladder mucosa has been shown to attenuate bleomycin-induced pulmonary fibrosis [23]. As a secreted product of the resident cells, ECM exhibits tissue-specific composition and functionality [33–35], suggesting that lung-derived ECM could be more effective than ECM derived from extra-pulmonary sources in protecting against lung damage.

Our decellularized ECM preparation is a unique fine particulate (3 μm or less) suspension/solution that promotes uniform distribution throughout the distal lung when delivered via airway instillation or nebulization. The detergent SDS is widely used to decellularize many tissues including lung [12]. Consistent with published reports [34,36], SDS disrupts cell membrane with complete removal of cellular nuclear material as confirmed by histology. The residual DNA amount was less than 50 ng/mg dry ECM weight, which is below the generally accepted level that might stimulate host immune response [12]. The decellularization process altered ECM structure and composition. Notably, GAG content was reduced, as has been shown by others [34,37]. GAGs contribute to tissue viscoelasticity, cell-matrix interactions and tissue remolding. Most GAGs are water soluble; therefore, they are easily washed away when exposed to detergents [38]. On the other hand, decellularized ECM is enriched in collagen compared to the native lung (Fig 2F). A previous report found an increased collagen content in decellularized whole lung ECM scaffold, which is associated with a lower compliance compared to the native lung [37]. Collagen is more resistant to SDS than non-collagen proteins. Because of a relatively large loss of total protein and relative retention of collagen, following decellularization collagen constituted a higher percentage of total proteins [37].

Pepsin is generally used for protein digestion and solubilization at room temperature [18,24,38]. When the digested ECM solution was neutralized by NaOH, diluted and passed through a 1.2 μm filter, the 1.6 mg/mL solution had the smallest particle diameter (< 1 μm). With increasing ECM concentration, particle aggregation was observed. To optimize uniform pulmonary delivery and maximal cellular uptake without excessive particle aggregation or loss during nebulization, we selected a final ECM concentration of 3.2 mg/ml. This preparation remained stable without significant changes in particle size after storage at 4°C.

Excessive generation of ROS is a key mechanism of cell damage and death in hyperoxia-induced ALI [39]. A variety of bioactive molecules exhibit cytoprotective effects on lung cells under oxidative stress, including α-Klotho [40], erythropoietin [41], epidermal growth factor-like domain-7 [10] and NF-κB [42]. In addition to single molecules or pathways, complex cocktails such as our lung ECM preparation also protect lung cells and lungs from oxidative apoptosis and damage to DNA, proteins and lipids. The complex components of our lung ECM including known cytoprotectors such as NF-κB and epidermal growth factor-like domains protein [10,42]. More detailed analysis of lung ECM currently underway may identify additional cytoprotective molecules.

Hyperoxia exposure is an established model of lung injury, and the cause of bronchopulmonary dysplasia in the developing lung of premature infants. The beneficial effects of ECM shown in adult rat lungs in this report may have translational value in the treatment of inflammatory lung disease. The two delivery methods (instillation or nebulization) and two levels of hyperoxia exposure (intermittent or continuous) show concordant responses. Both instillation and nebulization are effective routes of drug delivery to the lung [43–45]. Coarse particles may be instilled via a tracheal tube but its distribution in distal lung is uneven [44]. Nebulization promotes uniform ECM distribution in distal lung but limits the concentration of ECM in solution and the size of ECM microparticles. There may be ECM loss in the delivery circuit and the upper airway. In saline treatment group, continuo us or intermittent hyperoxia exposure elicited similar oxidative damage to DNA, protein and lipid. Compared to saline treatment, ECM delivered by instillation attenuated the alveolar septal thickening, apoptosis and oxidative damage to DNA protein and lipids during continuous hyperoxia. ECM delivered by nebulization showed similar mitigating effects on apoptosis and oxidative protein damage during intermittent hyperoxia while the smaller reductions in DNA and lipid oxidative damage were non-significant. The differences are likely related to differences in the severity of continuous vs. intermittent hyperoxia and in the delivery methods. Both delivery methods attenuated alveolar septal thickening and exudation. Nebulization is preferable for future development of our approach as it allows repetitive noninvasive delivery without anesthesia, and is the standard method for clinical drug delivery to the lung.

Mechanisms of ECM cytoprotection involve not only the actions of individual components but also their network interactions, and are the focus of intense investigation. Both stimulators and inhibitors of a given pathway may co-exist in the ECM cocktail and exert counter-balancing actions. We have established the feasibility of a novel lung protective strategy via exogenous decellularized ECM. A mechanistic exploration is beyond the scope of this *proof-of-concept* report, but is being pursued in our on-going studies. The magnitude of ECM-mediated cytoprotective effects is modest, at least partly because *in vivo* dose was restricted by the maximum concentration and volume of ECM solution/suspension that could be nebulized. On the other hand, broad balanced modulation of multiple metabolic pathways more closely mimics physiological reality whereas selective supra-physiologic manipulation of one or a few molecules might incur unbalanced responses causing adverse side effects. Compared to coarse ECM powder [23], a dilute fine suspension/solution should minimize potential immunological reactions with repeated administration.

## Conclusions

We manufactured and characterized a lung-specific decellularized ECM microparticle suspension/solution designed for inhalation delivery, demonstrated its efficacy in ameliorating pulmonary oxidant damage, and identified several bioactive components of this complex mixture that could have mediated the observed effects. These results support the use of decellularized ECM as a novel lung protection strategy, and direct further investigation to clarify its mechanisms of action. Future studies will aim to identify additional ECM components and their interactions, standardize the preparation, optimize dose-response and immune profiles following repeated administration, and examine a possible role for ECM-mediated potentiation of conventional pharmacological agents used in the treatment of acute lung injury.

## References

[1] Rubenfeld GD, Caldwell E, Peabody E, Weaver J, Martin DP, Neff M, et al Incidence and outcomes of acute lung injury. N Engl J Med. 2005; 353 (16): 1685–93. 10.1056/NEJMoa050333 16236739

[2] Johnson ER, Matthay MA. Acute lung injury: epidemiology, pathogenesis, and treatment. J Aerosol Med Pulm Drug Deliv. 2010; 23 (4): 243–52. 10.1089/jamp.2009.0775 20073554 PMC3133560

[3] Chow CW, Herrera Abreu MT, Suzuki T, Downey GP. Oxidative stress and acute lung injury. Am J Respir Cell Mol Biol. 2003; 29 (4): 427–31. 10.1165/rcmb.F278 14500253

[4] Xu D, Guthrie JR, Mabry S, Sack TM, Truog WE. Mitochondrial aldehyde dehydrogenase attenuates hyperoxia-induced cell death through activation of ERK/MAPK and PI3K-Akt pathways in lung epithelial cells. Am J Physiol Lung Cell Mol Physiol. 2006; 291: L966–L75. 10.1152/ajplung.00045.2006 16782756

[5] Mach WJ, Thimmesch AR, Pierce JT, Pierce JD. Consequences of hyperoxia and the toxicity of oxygen in the lung. Nurs Res Pract. 2011; 2011: 260482 10.1155/2011/260482 21994818 PMC3169834

[6] Barker GF, Manzo ND, Cotich KL, Shone RK, Waxman AB. DNA damage induced by hyperoxia. Am J Respir Cell Mol Biol. 2006; 35 (3): 277–88. 10.1165/rcmb.2005-0340OC 16574945 PMC2643280

[7] Budinger GRS, Mutlu GM, Urich D, Soberanes S, Buccellato LJ, Hawkins K, et al Epithelial cell death is an important contributor to oxidant-mediated acute lung injury. Am J Respir Crit Care Med. 2011; 183 (8): 1043–54. 10.1164/rccm.201002-0181OC 20959557 PMC3086743

[8] Jain D, Atochina-Vasserman EN, Tomer Y, Kadire H, Beers MF. Surfactant protein D protects against acute hyperoxic lung injury. Am J Respir Crit Care Med. 2008; 178 (8): 805–13. 10.1164/rccm.200804-582OC 18635887 PMC2566792

[9] Thébaud B, Ladha F, Michelakis ED, Sawicka M, Thurston G, Eaton F, et al Vascular endothelial growth factor gene therapy increases survival, promotes lung angiogenesis, and prevents alveolar damage in hyperoxia-induced lung injury: evidence that angiogenesis participates in alveolarization. Circulation. 2005; 112 (16): 2477–86. 10.1161/CIRCULATIONAHA.105.541524 16230500

[10] Xu D, Perez RE, Ekekezie II, Navarro A, Truog WE. Epidermal growth factor-like domain 7 protects endothelial cells from hyperoxia-induced cell death. Am J Physiol Lung Cell Mol Physiol. 2008; 294: L17–L23. 10.1152/ajplung.00178.2007 17934064

[11] Cepkova M, Matthay MA. Pharmacotherapy of acute lung injury and the acute respiratory distress syndrome. J Intensive Care Med. 2006; 21 (3): 119–43. 10.1177/0885066606287045 16672636 PMC2765330

[12] Crapo PM, Gilbert TW, Badylak SF. An overview of tissue and whole organ decellularization processes. Biomaterials. 2011; 32 (12): 3233–43. 10.1016/j.biomaterials.2011.01.057 21296410 PMC3084613

[13] Ott HC, Clippinger B, Conrad C, Schuetz C, Pomerantseva I, Ikonomou L, et al Regeneration and orthotopic transplantation of a bioartificial lung. Nat Med. 2010; 16 (8): 927–33. 10.1038/nm.2193 20628374

[14] Ren X, Moser PT, Gilpin SE, Okamoto T, Wu T, Tapias LF, et al Engineering pulmonary vasculature in decellularized rat and human lungs. Nat Biotech. 2015; 33 (10): 1097–102. 10.1038/nbt.3354 26368048

[15] DeQuach JA, Mezzano V, Miglani A, Lange S, Keller GM, Sheikh F, et al Simple and high yielding method for preparing tissue specific extracellular matrix coatings for cell culture. PloS One. 2010; 5 (9): e13039 10.1371/journal.pone.0013039 20885963 PMC2946408

[16] Lee JS, Shin J, Park HM, Kim YG, Kim BG, Oh JW, et al Liver extracellular matrix providing dual functions of two-dimensional substrate coating and three-dimensional injectable hydrogel platform for liver tissue engineering. Biomacromolecules. 2014; 15 (1): 206–18. 10.1021/bm4015039 24350561

[17] Singelyn JM, Sundaramurthy P, Johnson TD, Schup-Magoffin PJ, Hu DP, Faulk DM, et al Catheter-deliverable hydrogel derived from decellularized ventricular extracellular matrix increases endogenous cardiomyocytes and preserves cardiac function post-myocardial infarction. J Am Coll Cardiol. 2012; 59 (8): 751–63. 10.1016/j.jacc.2011.10.888 22340268 PMC3285410

[18] Wolf MT, Daly KA, Brennan-Pierce EP, Johnson SA, Carruthers CA, D'Amore A, et al A hydrogel derived from decellularized dermal extracellular matrix. Biomaterials. 2012; 33 (29): 7028–38. 10.1016/j.biomaterials.2012.06.051 22789723 PMC3408574

[19] Mendez JJ, Ghaedi M, Steinbacher D, Niklason L. Epithelial cell differentiation of human mesenchymal stromal cells in decellularized lung scaffolds. Tissue Eng Part A. 2014; 20(11–12): 1735–46. 10.1089/ten.TEA.2013.0647 24393055 PMC4029048

[20] Jeffords ME, Wu J, Shah M, Hong Y, Zhang G. Tailoring material properties of cardiac matrix hydrogels to induce endothelial differentiation of human mesenchymal stem cells. ACS Appl Mater Interfaces. 2015; 7 (20): 11053–61. 10.1021/acsami.5b03195 25946697 PMC4684185

[21] Brown BN, Valentin JE, Stewart-Akers AM, McCabe GP, Badylak SF. Macrophage phenotype and remodeling outcomes in response to biologic scaffolds with and without a cellular component. Biomaterials. 2009; 30 (8): 1482–91. 10.1016/j.biomaterials.2008.11.040 19121538 PMC2805023

[22] Badylak SF, Valentin JE, Ravindra AK, McCabe GP, Stewart-Akers AM. Macrophage phenotype as a determinant of biologic scaffold remodeling. Tissue Eng Part A. 2008; 14 (11): 1835–42. 10.1089/ten.tea.2007.0264 18950271

[23] Manni ML, Czajka CA, Oury TD, Gilbert TW. Extracellular matrix powder protects against bleomycin-induced pulmonary fibrosis. Tissue Eng Part A. 2011; 17 (21–22): 2795–804. 10.1089/ten.tea.2011.0023 21797754 PMC3204203

[24] Singelyn JM, DeQuach JA, Seif-Naraghi SB, Littlefield RB, Schup-Magoffin PJ, Christman KL. Naturally derived myocardial matrix as an injectable scaffold for cardiac tissue engineering. Biomaterials. 2009; 30 (29): 5409–16. 10.1016/j.biomaterials.2009.06.045 19608268 PMC2728782

[25] Williams C, Quinn KP, Georgakoudi I, Black LD III. Young developmental age cardiac extracellular matrix promotes the expansion of neonatal cardiomyocytes in vitro. Acta Biomater. 2014; 10 (1): 194–204. 10.1016/j.actbio.2013.08.037 24012606 PMC3840040

[26] Gilbert TW, Freund JM, Badylak SF. Quantification of DNA in biologic scaffold materials. J Surg Res. 2009; 152 (1): 135–9. 10.1016/j.jss.2008.02.013 18619621 PMC2783373

[27] Woessner JF Jr. The determination of hydroxyproline in tissue and protein samples containing small proportions of this imino acid. Arch Biochem Biophys. 1961; 93 (2): 440–7. 13786180 10.1016/0003-9861(61)90291-0

[28] Petersen TH, Calle EA, Zhao L, Lee EJ, Gui L, Raredon MB, et al Tissue-engineered lungs for in vivo implantation. Science (New York, NY). 2010; 329 (5991): 538–41. 10.1126/science.1189345 PMC3640463 20576850

[29] Vlodavsky I, Folkman J, Sullivan R, Fridman R, Ishai-Michaeli R, Sasse J, et al Endothelial cell-derived basic fibroblast growth factor: synthesis and deposition into subendothelial extracellular matrix. Proc Natl Acad Sci U S A. 1987; 84 (8): 2292–6. 3470794 10.1073/pnas.84.8.2292 PMC304636

[30] Heremans A, Cassiman JJ, Van den Berghe H, David G. Heparan sulfate proteoglycan from the extracellular matrix of human lung fibroblasts. Isolation, purification, and core protein characterization. J Biol Chem. 1988; 263 (10): 4731–9. 2450875

[31] Gilpin SE, Guyette JP, Gonzalez G, Ren X, Asara JM, Mathisen DJ, et al Perfusion decellularization of human and porcine lungs: bringing the matrix to clinical scale. J Heart Lung Transplant. 2014; 33 (3):298–308. 10.1016/j.healun.2013.10.030 24365767

[32] Booth AJ, Hadley R, Cornett AM, Dreffs AA, Matthes SA, Tsui JL, et al Acellular normal and fibrotic human lung matrices as a culture system for in vitro investigation. Am J Respir Crit Care Med. 2012; 186 (9): 866–76. 10.1164/rccm.201204-0754OC 22936357 PMC3530219

[33] Wallis JM, Borg ZD, Daly AB, Deng B, Ballif BA, Allen GB, et al Comparative assessment of detergent-based protocols for mouse lung de-cellularization and re-cellularization. Tissue Eng Part C Methods. 2012; 18 (6): 420–32. 10.1089/ten.TEC.2011.0567 22165818 PMC3358122

[34] Jensen T, Roszell B, Zang F, Girard E, Matson A, Thrall R, et al A rapid lung de-cellularization protocol supports embryonic stem cell differentiation in vitro and following implantation. Tissue Eng Part C Methods. 2012; 18 (8): 632–46. 10.1089/ten.TEC.2011.0584 22404373 PMC3401389

[35] Merna N, Fung KM, Wang JJ, King CR, Hansen KC, Christman KL, et al Differential β3 integrin expression regulates the response of human lung and cardiac fibroblasts to extracellular matrix and its components. Tissue Eng Part A. 2015; 21(15–16): 2195–205. 10.1089/ten.TEA.2014.0337 25926101 PMC4528988

[36] O'Neill JD, Anfang R, Anandappa A, Costa J, Javidfar J, Wobma HM, et al Decellularization of human and porcine lung tissues for pulmonary tissue engineering. Ann Thorac Surg. 2013; 96 (3): 1046–55; discussion 55–6. 10.1016/j.athoracsur.2013.04.022 23870827 PMC4033908

[37] Price AP, England KA, Matson AM, Blazar BR, Panoskaltsis-Mortari A. Development of a decellularized lung bioreactor system for bioengineering the lung: the matrix reloaded. Tissue Eng Part A. 2010; 16 (8): 2581–91. 10.1089/ten.TEA.2009.0659 20297903 PMC2947435

[38] Wu J, Ding Q, Dutta A, Wang Y, Huang Y-H, Weng H, et al An injectable extracellular matrix derived hydrogel for meniscus repair and regeneration. Acta Biomater. 2015; 16: 49–59. 10.1016/j.actbio.2015.01.027 25644450

[39] Zaher TE, Miller EJ, Morrow DMP, Javdan M, Mantell LL. Hyperoxia-induced signal transduction pathways in pulmonary epithelial cells. Free Radic Biol Med. 2007; 42 (7): 897–908. 10.1016/j.freeradbiomed.2007.01.021 17349918 PMC1876680

[40] Ravikumar P, Ye J, Zhang J, Pinch SN, Hu MC, Kuro-o M, et al α-Klotho protects against oxidative damage in pulmonary epithelia. Am J Physiol Lung Cell Mol Physiol. 2014; 307: L566–L75. 10.1152/ajplung.00306.2013 25063799 PMC4187038

[41] Ravikumar P, Menon JU, Punnakitikashem P, Gyawali D, Togao O, Takahashi M, et al Nanoparticle facilitated inhalational delivery of erythropoietin receptor cDNA protects against hyperoxic lung injury. Nanomedicine. 2015; 12(3): 811–21. 10.1016/j.nano.2015.10.004 26518603 PMC4809756

[42] Franek WR, Morrow DMP, Zhu H, Vancurova I, Miskolci V, Darley-Usmar K, et al NF-κB protects lung epithelium against hyperoxia-induced nonapoptotic cell death–oncosis. Free Radic Biol Med. 2004; 37 (10): 1670–9. 10.1016/j.freeradbiomed.2004.08.007 15477018

[43] Okamoto T, Tang X, Janocha A, Farver CF, Gladwin MT, McCurry KR. Nebulized nitrite protects rat lung grafts from ischemia reperfusion injury. J Thorac Cardiovasc Surg. 2013; 145 (4): 1108–16.e1. 10.1016/j.jtcvs.2012.04.006 23142117

[44] Liu F, Li W, Pauluhn J, Trübel H, Wang C. Lipopolysaccharide-induced acute lung injury in rats: Comparative assessment of intratracheal instillation and aerosol inhalation. Toxicology. 2013; 304:158–66. 10.1016/j.tox.2012.12.020 23313377

[45] Struber M, Fischer S, Niedermeyer J, Warnecke G, Gohrbandt B, Gorler A, et al Effects of exogenous surfactant instillation in clinical lung transplantation: a prospective, randomized trial. J Thorac Cardiovasc Surg. 2007; 133 (6): 1620–5. 10.1016/j.jtcvs.2006.12.057 17532965

